# INNOVATIVE APPROACHES TO MENTAL HEALTH SERVICES FOR DIVERSE COMMUNITY OLDER ADULTS

**DOI:** 10.1093/geroni/igad104.2873

**Published:** 2023-12-21

**Authors:** Tobi Abramson, Jo Anne Sirey, Jacquelin Berman

**Affiliations:** NYC Department for the Aging, Greenvale, New York, United States; Weill Cornell Medicine, Ithaca, New York, United States; NYC Department for the Aging (NYC Aging), New York City, New York, United States

## Abstract

Addressing the triple stigma (age, mental health, race) faced by older adults in need of mental health services has necessitated innovations that move the service provision needle. Co-located services within community settings, mental health interventions addressing those with nutritional needs, and victims of elder abuse and elder crime benefit from innovative services. The following session will highlight research outcomes from innovative treatment models: (a) In a sample of 138 diverse individuals participating in PROTECT (a brief behavioral intervention for late-life depression among elder abuse/crime victims) showed 5.15 point reductions in PhQ-9 scores across the sample and found video-delivery was non-inferior to traditional in-person therapy. Those receiving video therapy completed treatment faster, with more rapid rate of symptom reduction. (b) Do More Feel Better (a 9-week peer-led manualized intervention for those with depression and nutritional needs) found 78% experienced clinically significant reduction in depressive symptoms and over two-thirds (66%) reported satisfaction with the program. (c) DGMH (mental health services co-located in older adult centers) data indicate that of the 4,118 clients assessed, 3,373 endorsed at least one mental health need (81.9%), significantly higher than the mental health literature indicates. Over 2,133 clients have been treated in 67,597 sessions since program inception in 2018. Within 3-months of treatment, 60.8% and 52% showed clinically significant improvements in depression and anxiety, respectively. A third reported decreased loneliness and 39.4% indicated feeling less socially isolated. Further data will be presented (demographic, racial, ethnic differences). Data supported efficacy lend support to innovative mental health treatment approaches.

